# Alternative Clinical Practice During the COVID-19 Pandemic: A Qualitative Study of Nursing Students’ Potential Loss of Experience

**DOI:** 10.1177/23779608231178066

**Published:** 2023-05-30

**Authors:** Bente Dale Malones, Marita Christina Susanne Melbye, Trude Fløystad Eines, Johanne Alteren

**Affiliations:** 1 Faculty of Health Sciences and Social Care, 5562 Molde University College, Molde, Norway; 2 Faculty of Health Sciences and Social Care, 5562 Molde University College, Kristiansund, Norway

**Keywords:** autonomy in nursing, clinical practice, nursing education, COVID-19 lockdown, learning outcomes

## Abstract

**Introduction:**

During the COVID-19 pandemic, the lockdown of nursing institutions changed nursing students’ learning conditions in clinical practice. They were removed from their ordinary clinical practice and isolated in their homes for one week before resuming an alternative clinical practice. Owing to the changed learning conditions, nursing students had to readjust and find new and different solutions to manage their own learning.

**Objectives:**

This study aimed to investigate the impact of removing first-year nursing students from clinical practice during the COVID-19 pandemic on their perceived learning and development.

**Methods:**

A qualitative descriptive and interpretative design based on group and individual interviews was used. Eleven first-year nursing students from a university college in Norway participated in the study. They were interviewed from their homes, after completing their first 10-week clinical practice. Three group interviews and two individual interviews were recorded using the digital live video aid Zoom and analyzed using systematic text condensation.

**Results:**

The main theme “loss of experience,” summarized how the nursing students experienced their first clinical practice during the COVID-19 lockdown. The nursing students shifted from a predictable to an unpredictable clinical practice without supervision from the nurse supervisor or the nurse educator. The organization and planning of the practice were left to the students, who bore the responsibility of achieving the learning outcomes. Three categories were identified: unpredictable learning situations, compensating learning activities, and achieving learning outcomes.

**Conclusions:**

Nursing students faced difficulties in their first clinical practice during the COVID-19 lockdown, which made a challenging study situation. Too much autonomy and responsibility for their own learning reinforced a loss of experience. The uncertainty of the study situation potentially harmed their professional development, learning outcomes, and self-efficacy, especially concerning basic nursing skills.

## Introduction

The COVID-19 pandemic has affected frontline personnel and clinical management in health services, systems, and communities worldwide (World Health Organization [WHO], 2020). In addition, it has altered the learning process among nursing students in higher education institutions, severely affecting the learning environment in clinical practice in Norway and elsewhere (Ministry of Education and Research, 2020; WHO, 2020).

When the severe acute respiratory syndrome-coronavirus-2 virus began to spread globally, the first step taken by the Norwegian government was to shut down all academic institutions. In March 2020, all Norwegian nursing education institutions removed nursing students from clinical practice at hospitals and municipal health organizations (Ministry of Education and Research, 2020). The students were deprived of supportive learning environments in clinical practice, typically involving nurse supervisors, nurse educators, and learning activities such as guided reflection, prebriefing, and debriefing (Hayter & Jackson, 2020). Consequently, nursing students experienced a lack of training in patient care and guidance on various practical skills (Holmsen, 2010; Houghton et al., 2013; Olsen & Knudsen, 2015). They also suffered from a lack of feedback from nurse supervisors on the practice of nursing, which could improve learning outcomes in clinical practice.

Universities and university colleges had to make ethical assessments before deciding on the lockdown (Ministry of Education and Research, 2020). The value of the nursing students’ educational progress was weighed against the risk for and burden on patients, nursing students, and health professionals. The primary reason for taking this step was to prevent nursing students from exacerbating the spread of the infection (Ministry of Education and Research, 2020). After the decision was announced, nursing students had to return to their hometowns on very short notice, unaware of what this change would imply for their educational progress (Utvær et al., 2021).

## Review of Literature

Research concerning clinical practice during the COVID-19 pandemic contends that nursing students have experienced mental challenges and significantly increased stress and anxiety associated with fear of infection (Casafont et al., 2021; Heilferty et al., 2021; Hernández-Martínez et al., 2021; Jackman et al., 2020; Lourenco Goncalves et al., 2022; Lovrić et al., 2020; Martin-Delgado et al., 2021; Wallace et al., 2021). Although Wallace et al. (2021) found that the stress experienced by nursing students during the COVID-19 pandemic increased their motivation, some students reported that excessive stress has contributed to feelings of inadequacy and reduced nursing practice (Heilferty et al., 2021; Wallace et al., 2021). Support from family and friends seems crucial for reducing discomfort and anxiety among nursing students and helping them master their emotions through their nursing education (Casafont et al., 2021; Heilferty et al., 2021; Jowsey et al., 2020). Owing to the lack of clinical practice during the pandemic, nursing students have shown concern about the absence of interaction and communication with patients and practical learning activities (Casafont et al., 2021). However, nursing students highlight how nursing educators have adapted the curriculum, exam literature, teaching methods, and learning outcomes and adjusted the evaluation criteria (Lovrić et al., 2020) to suit the new circumstances.

Previous research has recorded and analyzed the COVID-19 pandemic-related increase in stress and anxiety among nursing students related to lack of training in patient care, loss of professional development, and fear of infection. Nevertheless, there is a lack of knowledge regarding nursing students’ experiences in clinical practice during the pandemic. This lack of knowledge pertains especially to the learning environments in the students’ first clinical practice, with no previous experience with supervised practice. In addition, previous research on this topic has mainly been quantitative. Very few qualitative studies have been published describing nursing students’ experiences of how COVID-19 has altered their learning process in higher education. To address this research gap, this study aimed to investigate the impact of removing first-year nursing students from clinical practice during the COVID-19 pandemic on their perceived learning and development.

## Methods

### Design

This study adopted a qualitative descriptive and interpretative design based on group and individual interviews. The proposed method was deemed suitable for phenomena that could be counted or measured but needed to be interpreted and analyzed considering the context, content, people, and consequences (Malterud, 2001; Polit & Beck, 2021). Group interviews allowed for sharing and discussing within the group, while individual interviews contributed to developing in-depth knowledge of the topic. Through group and individual interviews, the researchers gathered rich and detailed descriptions and inside knowledge about the issue at stake from experienced individuals (Polit & Beck, 2021). The Consolidated Criteria for Reporting Qualitative Studies guided the study's reporting.

### Research Question

How did the nursing students experience an alternative clinical practice during the COVID-19 pandemic?

### Sample

First-year nursing students (*n*  =  117) from a bachelor's degree at a university college in Norway were invited to participate in this study. The bachelor's degree is a full-time course with a total of 180 ECTS credits over three years (six semesters) and is equally divided between theoretical and clinical studies. Upon completion, students are awarded a Bachelor of Nursing and are qualified to practice as registered nurses in Norway.

### Inclusion Criteria

The inclusion criteria were nursing students who had completed their first 10-week clinical practice in a nursing home, care center, home nursing environment, or psychiatric ward during the COVID-19 lockdown in the spring of 2020. To complete their first 10-week clinical practice, the nursing students contacted healthcare institutions in their home environment with a request for an internship. The nursing students were given a nonsupervised internship. The nurse educator and nurse supervisor were exempted from supervision in the clinic owing to the risk of infection and increased workload.

The nurse educators provided nursing students with information about the study's purpose and procedures and discussed relevant ethical issues. In addition, the nurse educators and supervisors distributed a written consent form, inviting nursing students to participate via the digital learning platform Canvas. The participating nursing students signed a consent form.

### Data Collection

Data were collected using semistructured interviews, which included three group interviews with three participants in each group and two individual interviews. The researchers developed and followed an interview guide based on previous research and theory. The interview questions focused on how the nursing students’ clinical practice was organized, the collaboration between nursing students, nurse supervisors, and nurse educators, the use of digital tools in the learning process, the possibility of achieving the required learning outcomes, and the future of nursing students. The researchers conducted the interviews approximately two weeks after the nursing students had completed their clinical practice.

To prevent the spread of COVID-19 among the participants and researchers, the interviews were recorded using the digital live video aid Zoom. The nursing students participated in the interviews from their homes. The study needed to collect rich enough data to improve the current understanding of the nursing students’ experience, irrespective of the amount of data or the number of sources (Polkinghorne, 2005). The first and second authors, who were also moderators and note-takers, facilitated the interviews. Each interview lasted between 45 and 75 min and was audio recorded, transcribed, and anonymized. The moderators used the last few minutes of each interview to summarize and validate the main findings with the participants. During data collection and analysis, the researchers recorded (in writing) their reflections for use in the following interviews and analyses.

### Ethical Considerations

Ethical approval was granted by the Norwegian Social Science Data Services. The participants received verbal and written information about voluntary participation and could withdraw from the study at any time without consequences and having to explain why; however, no one withdrew. During the interviews, the participants’ faces and names were hidden behind the black screen in Zoom to safeguard their anonymity.

### Data Analysis

The interview data were analyzed using the qualitative content analysis method proposed by Graneheim and Lundman (2004). Based on inductive reasoning (Corbin & Strauss, 2015; Graneheim et al., 2017), the authors adopted an open-minded analysis approach to identify meaningful subjects and interpret the nursing students’ experiences. This content analysis aimed to produce a condensed yet extensive description and understanding of the issue at stake (Lindgren et al., 2020).

First, all researchers read each transcribed interview several times to obtain an overall understanding of its content. Second, the researchers divided the text into “meaning units,” namely, condensed versions capturing the meaning of the full text. Each meaning unit comprised words and sentences that contained aspects related to each other. Third, the researchers further condensed the meaning units and labeled them with codes in relation to the context (Graneheim & Lundman, 2004). Next, the researchers classified the meaning units into subcategories based on interpretation similarities and differences. A subcategory consisted of similarly interpreted condensed meaning units. Finally, the researchers formulated the categories to describe the research's main theme at the latent level.

The analysis implied a back-and-forth process between the whole and parts of the text (Graneheim & Lundman, 2004). The research team of four people met several times to discuss the results and reach a consensus regarding the text's meaning (Graneheim & Lundman, 2004; Graneheim et al., 2017). When no new information arose from rereading the transcriptions, the researchers considered data saturation achieved (Polkinghorne, 2005; Saunders et al., 2018). The categories are presented in the “Results” section, with quotations grounded in the nursing students’ experiences and representative of the participants’ responses.

## Results

### Sample Characteristics

Eleven first-year nursing students agreed to participate in the study. The nursing students’ (nine female and two male) ages ranged from 19 to 30 years old.

### Research Question Results

From the data analysis, three themes emerged. The main theme, “loss of experience,” summarized how the nursing students experienced their first clinical practice during the COVID-19 lockdown. The nursing students shifted from a predictable to an unpredictable clinical practice without supervision from the nurse supervisor or the nurse educator. Their clinical practice became alternative and included unpredictable learning situations and compensating learning activities involving various practical and theoretical tasks. The organization and planning of the practice were left to the students, who bore the responsibility of achieving the learning outcomes. Contact with the nurse educator and fellow students only occurred through Zoom meetings organized by the university college once a week. The researchers identified the following three categories of nursing students’ experiences in the data analysis: (a) unpredictable learning situations, (b) compensating learning activities, and (c) achieving learning outcomes.

### Unpredictable Learning Situations

After completing a week of ordinary clinical practice in nursing homes, nursing education institutions removed nursing students from clinical practice. After this announcement, the nursing students returned to their hometowns without clear expectations about the future of their education. Ordinary learning activities and supervision from the university college stopped during the first week of the lockdown. This supervision typically included at least three meetings with the nursing student, a defined nurse supervisor in the nursing home, and a nurse educator from the university college. These meetings focused on expectations and evaluations related to the nursing students’ learning outcomes, which, in a nursing home, related to the ability to provide basic and person-centered nursing care to patients based on nursing, scientific, and medical knowledge. In addition, students understood the nurse's role, management, and organization in relation to each patient and the nursing home health service.

The nursing students were isolated in their homes as they waited for a message from the university college about how they could continue their clinical practice. In the first days of the lockdown, information from the government was lacking. The lockdown was a completely new situation for the whole society.

After the first week of the lockdown, the nursing students began receiving different and unstructured information from various channels such as the media, the Norwegian government, and the university college. For example, the press wrote about the lockdown, the Norwegian government informed the population, and the university college posted messages on its website. The nursing students reported that they were exposed to an overload of information that led to feelings of chaos and insecurity. In addition, most of them elaborated on how fellow students interpreted the information differently, as confirmed by the students’ use of Facebook groups, through which they shared their interpretations, seeking clarity and straightforward answers. Consequently, this amplified the unpredictability of the learning environment. A nursing student reported her feeling of unclarity:

I felt confused when I had to stay home and just wait, while receiving many different messages in one day. (Student 6, Interview 4)

Another nursing student elaborated on how the information they received was contradictory:

At the beginning of the lockdown, we received different information from the university college depending on who we had talked to. For example, one person from the university college said that I should continue in clinical practice, another said that I had to go home. Different information was experienced as very confusing. (Student 3, Interview 2)

To meet the nursing students’ needs for a more predictable learning environment, the university college introduced weekly supervision and learning support using the digital platform Zoom. By using Zoom, students communicated with nurse educators and fellow students. The nursing students had different experiences with how comfortable they felt using Zoom. Some students were shy and embarrassed about sitting in front of a video screen. After a few meetings in small groups with other nursing students and the nurse educator, they became more comfortable talking and reflecting in front of the camera. One student reported:

The best experience from the weekly Zoom meetings was to see and listen to other nursing students’ thoughts and reflections on how they worked with the learning activities in the alternative clinical practice. (Student 9, Interview 3)

During the Zoom meetings, the nursing students and the nurse educator shared their experiences and feelings and discussed the learning environments. Typical topics in the meetings were discussions on clinical practice, how to achieve learning activities, and shared reflections on experiences in the alternative clinical practice. One student said:

I enjoyed the conversations and reflections in the Zoom meetings. I received help to reflect on how to integrate nursing theory into clinical practice. (Student 11, Interview 5)

Many of the nursing students also shared ideas about how to access and learn practical skills for patient care. They stated that supervision and support made them feel more confident.

### Compensating Learning Activities

In the first week of the lockdown, nursing education institutions organized compensatory learning activities that replaced ordinary learning activities. At the same time, the students waited for further clarifications on the continuation of their clinical practice. The main reason for providing compensatory learning activities was to prevent students from being delayed in completing their bachelor's degree. The compensatory learning activities corresponded to the number of hours of clinical practice in the ordinary study program.

Examples of compensatory learning activities were training in systematic clinical examination using the Airway, Breathing, Circulation, Disability, Exposé/Environment principles, and reflection notes regarding various case studies. The nurse educators developed case studies in which the students had to reflect on various topics such as hygiene, heart failure, and basic nursing. The students trained on family members and/or dolls. They wrote reports and made short videos documenting how they achieved the required skills. The results were submitted to the nurse educators, who assessed the reports and videos as approved or not.

Compensating learning activities were introduced to keep students active and focused on nursing. However, despite these efforts, all of the nursing students expressed discomfort regarding compensatory learning activities compared to actual clinical practice. They also stated that they were unsure of what compensatory learning activities the university college expected them to accomplish, as expressed by one of the nursing students:

I was worried about all the writing assignments I had to do. I did not understand what the university college expected me to do. My nurse educator supported me very well during this period in the weekly Zoom meetings. (Student 7, Interview 3)

Some nursing students found it challenging to handle compensatory learning activities compared to training in an actual clinical practice situation. They began losing courage and motivation when the clinical practice only consisted of compensatory learning activities.

### Achieving Learning Outcomes

After approximately two weeks of lockdown, the nursing students received information on how to continue their clinical practice, defined as an alternative practice. They were required to organize their own clinical practice. Before the pandemic, they would follow a 10-week practice. In contrast, the students were not even assigned a nursing supervisor owing to the clinic's risk of infection and increased workload. The nursing students were left to their own devices and charged with the responsibility of achieving the learning outcomes. One of them stated:

I had to find an alternative clinical practice near my home that could compensate for the ordinary clinical practice. It was challenging not to know if I would be able to access an internship so that I could continue as a nursing student. (Student 3, Interview 1)

They described this situation as demanding, especially for those who did not know anyone in the healthcare system in the local community. All the nursing students expressed concern about not developing the expected knowledge and skills in alternative clinical practice. One of them reported her concerns:

I could not complete the clinical practice in nursing homes as described in the curriculum. In my alternative clinical practice, I did not have a nurse supervisor who guided me towards achieving the learning outcomes. I experience that I have not acquired sufficient knowledge and skills in this clinical practice. (Student 2, Interview 3)

Usually, students develop knowledge of and skills in, for example, basic nursing, hygienic principles, observations, assessment of patients’ disease symptoms, and vital signs such as blood pressure measurement, heart rate, temperature, and respiratory rates. These students develop these experiences in close contact with patients. After the COVID-19 outbreak, close contact with patients became impossible, and nursing students began worrying about how they would achieve the learning outcomes. As one of the nursing students stated:

Alternative clinical practice without a nurse supervisor was not a good option for me. I felt alone without supervision. No one assessed whether I had achieved the learning outcomes during this period. (Student 4, Interview 2)

The nursing students often reported that they felt alone and were uncertain about how to best organize the learning activities without a nurse supervisor despite the weekly Zoom meetings with the nurse educator. As one nursing student elaborated:

I’m a shy person, and I had to push myself. I was uncomfortable and worked hard to achieve learning outcomes almost without supervision. (Student 11, Interview 5)

They wondered how the university college could assess whether they had achieved the learning outcomes. Some nursing students began losing motivation and described the new modality for achieving learning outcomes as “difficult to cope with.” In contrast, some of them liked the alternative clinical practice's flexibility. As one of the nursing students reported:

I learned a lot in the alternative clinical practice. The corona pandemic (COVID-19) was almost like an acute situation. I just had to participate, so I think I was both lucky and unlucky to be in clinical practice those days. (Student 8, Interview 3)

They emphasized the role of the national plan prepared by the Norwegian government, which provided guidelines for nursing students to continue their education instead of delaying their study progression. Nursing students reported that they were happy with the alternative way of moving forward instead of being forced to interrupt their studies.

At the same time, all of the students raised questions about whether the alternative practice was good enough and whether a lack of experience with patients could have consequences for their study's progression. As one of the nursing students stated:

I remember I was thinking a lot about my degree being delayed, and I was afraid because what if I did not reach expected learning outcomes. What if I must wait another year before I can continue my nursing education. (Student 2, Interview 1)

Another question concerned the level of learning outcomes the nurse educators expected them to achieve. Overall, the students felt they did not develop the knowledge and practical skills for the following clinical practice. They felt a lack of knowledge, especially concerning basic nursing skills.

## Discussion

This study highlighted how nursing students found it challenging to follow alternative instead of ordinary clinical practice. Alternative clinical practice required students to act independently, autonomously managing their alternative clinical practice one week into the COVID-19 lockdown. Autonomy in nursing is described as the “ability of the nurse to assess and perform nursing actions for patient care based on competence, professional expertise, and knowledge” (Nightingale College, 2022). It also refers to the registered nurses’ ability to think critically and take actions related to a patient's care (Nightingale College, 2022). In this study, the researchers sought to understand the extent to which the students autonomously managed to handle the alternative clinical practice.

The nursing students in this study ended up without any supervision and had to handle an unpredictable situation in an alternative clinical practice from their own point of view. As students are in a learning process, autonomy in nursing is taught through interaction with others, such as patients, nurses, fellow students, and educators (Little, 1991). Further, autonomy is the product of an interactive process in which the teacher gradually enlarges the scope of the learner's autonomy by gradually allowing them more control of the process and content of their learning (Little, 2007). Development of autonomy requires education and practice allowing the students to be in autonomy-promoting learning environments where the focus is on their learning needs, encouraging learner involvement, and where the learner is challenged in different theoretical and practical learning situations.

Alternative learning activities became a pedagogical approach to create a framework, structure, and learning process to keep the students’ focus. Through alternative learning activities, an autonomy-promoting learning environment can be striven for. The students in this study experienced a certain degree of support during the alternative learning activities, but the demand for autonomy and responsibility for their own learning was higher than they were prepared for, which for some students became too overwhelming. Autonomy in learning situations is important for professional learning and development (Little, 1991). The question is whether the demand for autonomy among the students was too high and whether the demand could come at the expense of the feeling of mastery and motivation. During the lockdown, the nursing students did not receive feedback from a nurse supervisor in patient situations. Lack of feedback in clinical practice may hinder nursing students’ motivation (Jowsey et al., 2020). According to Bandura's self-efficacy theory (Bandura, 1986), one's perceived self-efficacy is a judgment of one's capability to perform in a particular situation depending on feedback from other people. Lack of feedback put the nursing students in a situation where they did not know what competence they had and had not developed, how they were when it came to the learning outcomes, and how the competence could be applied. Lack of achievement of the learning outcomes, especially those concerning close contact with patients, was highlighted by the nursing students. For the nursing students, a lack of patient contact meant a lack of experiences of situations in which to practice nursing, an experience they defined as essential in becoming a nurse.

The nursing students in this study experienced loss of experience in clinical practice. At the same time, they showed commitment and effort, and they met the challenges and opportunities the situations presented. Little (2007) points out that it is in the person’s nature to be autonomous, to be proactive in exploring and responding to our environment, and to persist in following the agendas we set for ourselves. The nursing students supported each other in the weekly Zoom meetings. Encouraging and challenging each other are essential in autonomy-promoting learning environments. Through the weekly Zoom meetings, the students participated in and contributed to an autonomy-promoting learning environment, which was however limited to theory and practical solutions to challenges encountered in practice and the execution of reports and videos. Patient contact was a theoretical topic and responsibility was left to the students in these learning situations.

Students’ experiences of a lack of competence created uncertainty among them about the competence they had developed and areas in their competence that should be developed further. They had to rely on their own assessments related to the feedback from the teacher and fellow students in the weekly Zoom meetings. The feedback created a sense of security around the exercises of linking theory and practice, but also of insecurity when they knew that they had not reached the learning outcomes concerning expected knowledge and skills, based on the lack of close contact with patients. Little (1991) describes autonomy as a capacity for detachment, critical reflection, decision-making, and independent action and states that this capacity may be promoted in interaction with patients, nurses, fellow students, and educators. The students expressed the situation as an emergency with the consequence that they had to simply participate and take the opportunities that arose so that they could move forward instead of being forced to interrupt their education. Despite this, personalized opportunities and feedback provided to nursing students require supervision and collaboration with a nurse supervisor and academic nurse educators. Unfortunately, these options were unavailable during the COVID-19 lockdown, limiting nursing students’ experiences.

### Strengths and Limitations

This study described the nursing students’ views on how the pandemic influenced their clinical practice experiences and, as they could not receive regular supervision in their first clinical practice, clarified the consequences on students’ clinical experiences. However, despite its contributions, the present research suffered from some limitations. The findings of this qualitative study might not apply to nursing students worldwide, but they could, to some extent, be extended to similar contexts (Polit & Beck, 2021).

The number of participants in the study might limit the diversity of experiences and meanings assigned to the alternative practice during the lockdown. Nevertheless, participants in small-group interviews may be more active and engaged compared to groups with more participants (Eines & Thylen, 2012). Digital interviews could enhance long-distance participation and have been essential in this study to recruit participants who have returned to their hometowns. Digital interviews without video cameras might be a limitation, losing nonverbal communication and visual cues (Thunberg & Arnell, 2022). Despite these issues, digital interviews were the way to carry out this study during the COVID-19 pandemic. Eleven nursing students out of 117 participated in this study; hence, their opinions might not reflect the views of all the students.

During the interviews, the researchers asked the participants to verify the researchers’ understanding of their answers, thus increasing the study's trustworthiness. In addition, all the authors participated in the analysis. They discussed and agreed upon the condensed meaning units and categories at various levels, which ensured transparency and enhanced the credibility and validity of the study's findings. Moreover, the results were also reported using the participants’ own words.

### Implications for Practice

The study provides useful information for education institutions and clinical placements regarding the nursing students' learning process in their first clinical practice during the COVID-19 pandemic. This knowledge can lay the foundation for how to follow up with the students in their education. Nursing education institutions, nurse educators, and nurse supervisors may incorporate the findings of this study to design nursing education programs during a pandemic or similar situations in the future for students who are left without supervision and have to find solutions and organize their own learning.

## Conclusions

This study shows that nursing students faced difficulties in their first clinical practice during the COVID-19 lockdown. They experienced too much autonomy and responsibility for their clinical practice and uncertainty regarding whether they had achieved the expected learning outcomes. They lacked cooperation and feedback from a nurse supervisor. The sum of these moments created a study situation that potentially harms their development, learning outcomes, and self-efficacy. They reported experiencing too much responsibility, uncertainty, and a lack of cooperation and feedback.

After two years of alternative clinical practices and learning activities, educational institutions still lack an overview or awareness about the nursing students’ learning outcomes during their alternative clinical practices. The temporary regulations from the Ministry of Education and Research (2020) state that educational institutions must contrive to teach to avoid delays in nursing students’ learning. Nursing bachelor programs must promote resilience by coaching nursing students to find alternative ways to overcome challenges and reframe experiences from negative to positive. Therefore, further studies should investigate what nursing students have learned through clinical practices during the COVID-19 pandemic. Future research should also focus on how alternative clinical practices during the COVID-19 pandemic have potentially affected nursing students’ skills and the impact of the pandemic on teaching and learning.
